# A retrospective analysis of early vs late tracheostomy in ICU patients: comparison of severity, length of stay and outcome

**DOI:** 10.1186/2197-425X-3-S1-A943

**Published:** 2015-10-01

**Authors:** C Hadjilouca, G Minas, D Demetriou, P Georgiou, I Taliadoros, E Kyriakides, T Kyprianou

**Affiliations:** Intensive Care, Nicosia General Hospital, Nicosia, Cyprus; Nicosia General Hospital, Nicosia, Cyprus; St Georges University of London Medical Program at the University of Nicosia Medical School, Nicosia, Cyprus

## Introduction

Although recent studies suggest that early tracheostomy may improve some short-term clinical outcomes i.e. likelihood of weaning from mechanical ventilation and ICU length of stay, optimal timing of tracheostomy remains unclear, certainly for many ICU sub-groups.

## Objectives

To evaluate the clinical characteristics and outcomes of early tracheostomy (performed in up to 7 days after intubation) compared with late tracheostomy (performed in 8 or more days after intubation) in several subsets of an ICU consecutive patients' cohort.

## Methods

Data were collected from the PDMS (Phillips) and the PROSAFE (quality control and audit program) combined databases referring to all patients subjected to tracheostomy during the years 2013-2014, and analysed using the SPSS17.0. Variables' K-s distribution in the 2 groups was normal, thus parametric tests (t-test / ANOVA) were used to compare the 2 groups and determine outcome prognostic factors.

## Results

The study refers to a multidisciplinary ICU (Medical / Surgical / Neuro Surgery / Cardiac Surgery) that has more then 800 admissions per year with an average ICU mortality of 18%, mean LOS of 7 days and SMR of 0.92 for 2013. 328 patients (19% of total admissions of the period, only 4% of admissions with LOS< 24 hrs) received a tracheostomy. Early tracheostomy was performed in 239 patients (73%) and late tracheostomy in 89 patients (27%). Mean age: 58 and 53 years respectively. The two groups did not differ significantly as per the admission category (Emergency Surgical 27% VS 34% / Elective Surgical 6% VS 3% / Non-Surgical 67% VS 63%). Mean SAPSII and SOFA scores were higher in the late tracheostomy group (54.02 VS 47.99, p = 0.007) and (10.05 Vs 8.62, p = 0.03) respectively. ICU mortality was significantly higher in the late than in the early tracheostomy group (17/86, 20% VS 26/235, 11%, p = 0.043) though Hospital mortality was not significantly different 27% (64/235) VS 24% (21/86). Hospital stay was longer in the late tracheostomy group (28.4 VS 22.3 days, p = 0.007).

## Conclusion

In this cohort of consecutive, multidisciplinary ICU patients, those subjected to late tracheostomy were those of higher disease severity upon admission and had worse ICU outcome. The phenomenon seems to be diluted when it comes to Hospital mortality, a finding that needs further investigation and analysis into contributing factors and subgroups of patients.

